# Comparative Methods to Evaluate the Antioxidant Capacity of Propolis: An Attempt to Explain the Differences

**DOI:** 10.3390/molecules28124847

**Published:** 2023-06-19

**Authors:** Vanessa B. Paula, Letícia M. Estevinho, Susana M. Cardoso, Luís G. Dias

**Affiliations:** 1 Doctoral School, University of León (ULE), Campus de Vegazana, 24007 León, Spain; 2 Centro de Investigação de Montanha (CIMO), Instituto Politécnico de Bragança, 5300-252 Bragança, Portugal; leticia@ipb.pt (L.M.E.); ldias@ipb.pt (L.G.D.); 3 Laboratório Associado para a Sustentabilidade e Tecnologia em Regiões de Montanha (SusTEC), Instituto Politécnico de Bragança, Campus de Santa Apolónia, 5300-253 Bragança, Portugal; 4 Associated Laboratory for Green Chemistry of the Network of Chemistry and Technology LAQV-REQUIMTE, Department of Chemistry, University of Aveiro, 3810-193 Aveiro, Portugal; susanacardoso@ua.pt

**Keywords:** propolis, total phenolic compounds, antioxidant capacity, UHPLC-DAD-ESI-MS

## Abstract

Propolis is a natural product produced by bees that contains a complex mixture of compounds, including phenolic compounds and flavonoids. These compounds contribute to its biological activities, such as antioxidant capacity. This study analysed the pollen profile, total phenolic content (TPC), antioxidant properties, and phenolic compound profile of four propolis samples from Portugal. The total phenolic compounds in the samples were determined by six different techniques: four different Folin–Ciocalteu (F-C) methods, spectrophotometry (SPECT), and voltammetry (SWV). Of the six methods, SPECT allowed the highest quantification, while SWV achieved the lowest. The mean TPC values for these methods were 422 ± 98 and 47 ± 11 mg GAE/g sample, respectively. Antioxidant capacity was determined by four different methods: DPPH, FRAP, original ferrocyanide (OFec), and modified ferrocyanide (MFec). The MFec method gave the highest antioxidant capacity for all samples, followed by the DPPH method. The study also investigated the correlation between TPC and antioxidant capacity with the presence of hydroxybenzoic acid (HBA), hydroxycinnamic acid (HCA), and flavonoids (FLAV) in propolis samples. The results showed that the concentrations of specific compounds in propolis samples can significantly impact their antioxidant capacity and TPC quantification. Analysis of the profile of phenolic compounds by the UHPLC-DAD-ESI-MS technique identified chrysin, caffeic acid isoprenyl ester, pinocembrin, galangin, pinobanksin-3-*O*-acetate, and caffeic acid phenyl ester as the major compounds in the four propolis samples. In conclusion, this study shows the importance of the choice of method for determining TPC and antioxidant activity in samples and the contribution of HBA and HCA content to their quantification.

## 1. Introduction

Propolis is a natural mixture of resin from trees or shrubs, buds, leaves, bark, and plant exudates collected by bees, *Apis mellifera* L., to which they add small amounts of secretions from their salivary glands [1,2]. Its composition contains a complex mixture of compounds, including phenolic compounds (hydroxybenzoic and hydroxycinnamic acids) and flavonoids [3,4], which are believed to contribute to its biological activities, such as antibacterial [5], anti-inflammatory [5,6], antitumor [5,7], cytotoxic [8], and antioxidant activities [5], among others.

The determination of TPC is important for the evaluation of antioxidant activities, since studies have shown that there is a direct correlation between antioxidant capacity and the content of total phenolic compounds [9,10].

The most commonly used spectroscopic techniques for the determination of total phenolic content (TPC) are Fourier transform infrared spectroscopy (FT-IR), Raman spectroscopy, and the Folin–Ciocalteu (F-C) assay using UV–Vis spectroscopy [11,12]. The F-C method is an easy and economical technique for the measurement of total phenolic compounds and is suitable for routine laboratory use [13]. This colorimetric method requires the use of a reference substance (e.g., gallic acid) to measure the total concentration of phenolic hydroxyl groups in the plant extract [14]. The F-C technique is based on the reaction of phenolic compounds with the F-C reagent which, in the presence of sodium carbonate, forms a blue complex whose intensity is related to the concentration of phenols present in the sample [12,14,15]. In the F-C method, the reagents are prepared in water or polar organic solvents, which only allows the determination of hydrophilic phenols in a sample [13].

The determination of antioxidant capacity uses spectroscopic techniques such as the diphenyl-2-picrylhydrazyl (DPPH) assay, the chemiluminescence assay, the Trolox equivalent antioxidant capacity, the ferric-reducing power antioxidant assay (FRAP), and electron spin resonance (ESPR) [16]. The DPPH method only allows the determination of hydrophobic antioxidants, while FRAP is based on the reduction of transition metal ions, iron, and copper [9].

Alternatives to traditional methods for determining the TPC and antioxidant properties of phenolic compounds are electrochemical methods [3,17]. Cyclic voltammetry (CV), differential pulse voltammetry (DPV), and square wave voltammetry (SWV) techniques have been successfully used to detect phenolic compounds (phenolic acids and flavonoids) in a variety of aqueous and non-aqueous solutions [3,18]. The advantage of electrochemical techniques is that they allow rapid, simple, and inexpensive determinations and, in some cases, allow measurements in the presence of colouring or masking compounds that may interfere with measurements made by other techniques, e.g., spectrophotometry [19,20].

Several analytical methods can be used to characterise and identify the phenols present in propolis. Techniques such as high-performance liquid chromatography (HPLC) [21], capillary electrophoresis (CE) [22], chemiluminescence (CL) [23], ultra-high-resolution liquid chromatography–tandem mass spectrometry (UHPLC-MS/MS) [24], liquid chromatography coupled to mass spectrometry (LC-MS-MS) [25,26], and gas chromatography–mass spectrometry (GC-MS) [26,27] can be used. However, the most widely used is ultra-high-performance liquid chromatography coupled to triple quadrupole mass spectrometry (UHPLC-QqQ-MS/MS) due to its high sensitivity, selectivity, and high throughput [28]. The phenolic compounds detected in propolis samples were hydroxybenzoic acids (HBAs) and hydroxycinnamic acids (HCAs), as well as protocatechic, gentic, p-coumaric, ferulic, and caffeic acids [3,29]. Flavonoid aglycones of flavones, flavonols, flavonones, and chalcones are also present in propolis extracts, including pinobanksin, quercetin, apigenin, t-cinnamic acid, luteolin, chrysin, pinocembrin, galangin, kaempferol, and pinostrobin [3,30].

The aims of this work were to study the profile of phenolic compounds of four ethanolic propolis extracts collected from different regions of Portugal using the UHPLC-DAD-MS^n^ method; comparison of different analytical methods for TPC and antioxidant capacity; correlation of these analytical methods with the content of HBAs, HCAs, and FLAVs quantified in propolis samples; pollen analysis to determine the floral origin of propolis samples. The novelty of this work was the attempt to correlate the methods for quantifying TPC and antioxidant capacity with the HBA, HCA, and FLAV contents quantified in propolis samples, which allowed us to explain the differences between the methods. Besides contributing to standardisation of procedures, this may increase confidence in the assessment of the characteristics of propolis samples, ensuring their quality and efficacy in different applications.

## 2. Results

### 2.1. Pollinic Analysis

The results of the analysis of the pollen profile of propolis allow inferring its floral origin. The main pollen types and the percentage of the number of pollen grains counted in each sample are shown in Table 1. The pollen profile considering the main pollens included by ten pollen species: *Populus* sp., *Trifolium repens*, *Cistus ladanifer*, *Quercus* sp., *Pinus nigra*, *Leontodon* sp., *Castanea sativa*, *Euphorbia* sp., *Echium vulgare*, and *Olea europacea*. The four propolis samples have quite similar botanical origins. However, the predominant pollen types were different: *Trifolium repens*, Braga sample (25.6 ± 0.1%), *Populus* sp., Lousã and Macedo samples (51.5 ± 1.5% and 20.2 ± 0.2%, respectively), and *Castanea sativa*, Montesinho sample (35.0 ± 2.0%). All samples have high percentages of *Populus* sp. (percentages ranging from 51.5 ± 1.5% to 20.2 ± 0.2%). Only the sample from Lousã had a pollen predominance higher than 45%, which is classified as dominant pollen. All the other samples have only secondary pollen.

### 2.2. Total Phenolic Compounds

#### 2.2.1. Quantification of Total Phenolic Compounds with Six Methods

The TPC of the samples was determined spectrophotometrically (F-C methods and spectrophotometric method) and electrochemically (square wave voltammetry method). For the determination of total phenolic compounds, a calibration line with gallic acid (GA) as standard was established for each method. For the F-C, spectrophotometric, and SWV methods, the concentrations used for the calibration curve were 10–800, 20–240, and 20–280 mg/L, respectively. Square wave voltammograms were recorded in the range of +0.1 to +0.9 V. The results were expressed as mg gallic acid equivalents per g of sample (mg GAE/g) and are presented in Table 2.

The Macedo sample had higher levels of TPC, except for the SWV method. The TPC values obtained from the Lousã sample were the lowest for all six quantification methods examined in the study. Among these methods, the SWV method showed the lowest quantification with values ranging from 43 to 60 mg GAE/g extract, while the SPECT method showed the highest with values ranging from 263 to 503 mg GAE/g extract. The mean TPC values obtained by SPECT were approximately 2.8 times higher than those of the other methods studied. Of the four methods using the F-C reagent, the F-C4 method gave the lowest TPC results, while the F-C3 method gave the highest overall results.

A two-factor ANOVA without interaction was performed to check the differences between propolis samples (factor 1, four samples) and TPC quantification (factor 2, six methods). For both factors (factor 1: *p*-value < 0.001; factor 2: *p*-value < 0.001) there were significant differences in at least one of the means tested. Overall, the data show homogeneity of variances between factor 1 and factor 2 (Levene test, *p*-value = 0.331) and a distribution close to normality (Shapiro–Wilk test, *p*-value = 0.047). The ANOVA model was significant (*p*-value < 0.001) and has an RSE of 31.94, a DF of 63, and an R^2^ of 0.9474, indicating that the model accounts for 94.74% of the variability within the experimental data.

The result of the statistical analysis of the TPC concentration data showed that the samples from Montesinho and Braga were statistically similar (210 ± 133 and 209 ± 135 mg GAE/g extract, respectively) and different from the samples from Macedo and Lousã, which were statistically different (238 ± 145 and 113 ± 74 mg GAE/g extract, respectively). As for the methods used for TPC analysis, F-C1 and F-C4 were found to be similar, as were F-C2 and F-C3. Spectrophotometric and voltammetric methods gave statistically different results (MSerror = 1020; DF = 63). The mean TPC values in ascending order for the voltammetric, F-C4, F-C1, F-C2, F-C3, and spectrophotometric methods were 47 ± 11, 123 ± 33, 134 ± 34, 204 ± 55, 221 ± 73, and 422 ± 98 mg GAE/g sample, respectively.

**Figure 1:**
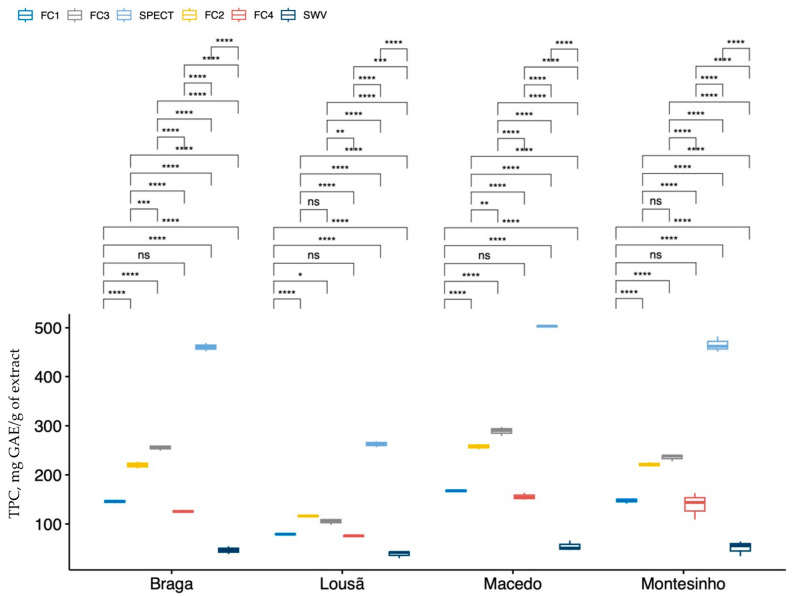
Box plots of each propolis sample compared with the means of the TPC methods. ns—not significant; the significance levels are translated by the following symbols: **** (*p*-value < 0.0001); *** (*p*-value < 0.001); ** (*p*-value < 0.01); * (*p*-value < 0.05); ns (*p*-value > 0.05).

Box plots were made for each sample and the means of each group were compared to see if there were significant differences. The results of these tests are depicted on the plots. This approach provided a clear visualisation of the data, making it easier to draw conclusions about the differences between the samples and the analytical methodologies (Figure 1).

Figure 1 shows that some TPC methods of analysis were significantly different from each other, confirming the results of the statistical analysis of the ANOVA, which shows that the F-C1 and F-C4 methods were indeed statistically equal, as were F-C2 and F-C3. The SPECT and SWV methods were statistically different, with higher and lower TPC values, respectively, in all samples. Overall, the behaviour of the different methods for the four propolis samples was similar. For the Lousã sample, the four F-C methods and the voltammetric method are close, with the spectrophotometric method standing out with higher values (on average 3.1 times higher than the other methods).

#### 2.2.2. Correlation of TPC with HBA, HCA, and FLAV

For the estimation models, the results of independent variables (HBA, HCA, and FLAV) reported in a previous work of our team [31] and the dependent variables (methods of analysis of the concentration of total phenolic compounds) were used in the logarithmic form (results presented in several orders of magnitude). The results of the estimation models obtained, as well as the values of RSE, R^2^, and *p*-value of method significance, are presented in Table 3.

Estimation models were of the type:

log([total phenolics]) = b + a1 × log([HBA]) + a2 × log([HCA]) + a3 × log([FLA])(1)

Table 3 reveals that the presence of HBA compounds in propolis samples does not have a significant impact on the quantification of TPC; however, it may have a negative influence on the F-C2 method. As for the HCA compounds, only the F-C2 and F-C3 methods showed a positive influence on the quantification of TPC, while the other methods did not show any significance. The presence of flavonoids showed a positive effect on quantifying TPC in four methods, while the F-C3 and SWV methods did not show any significance.

**Figure 2:**
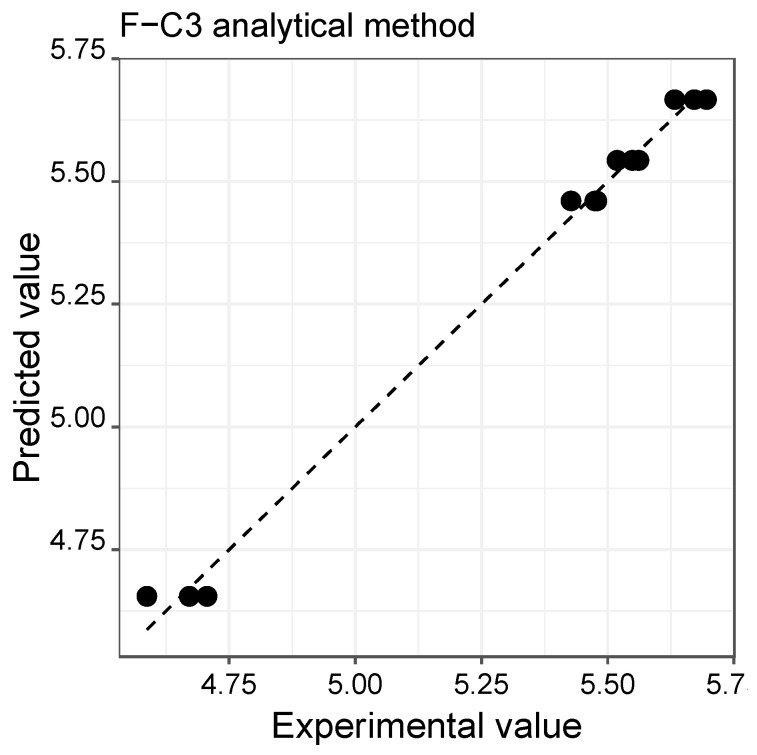
Linear relationship between the total phenolic content results (mg GAE/g of extract) obtained from the model estimation and the F-C3 analytical method.

Figure 2 shows, as an example, the plot of the data obtained by the estimation model about the experimental values obtained by the F-C3 method, verifying an acceptable linear fit (significant estimation model, *p*-value < 0. 001; slope = 0.992 ± 0.028 close to 1; intercept = 0.04 ± 0.15 close to 0; R^2^ = 0.992 close to 1), reflected in the low value of the RSE (0.039) and the acceptable closure coefficient (0.9920) obtained in the estimation model.

### 2.3. Antioxidant Capacity

#### 2.3.1. Estimation of the Antioxidant Capacity

In this study, four different methods were used to measure the antioxidant capacity of propolis samples collected from different geographical origins and L-ascorbic acid (positive control). The tests performed were a radical scavenging assay (DPPH) and three reducing power assays using iron (III) (FRAP, MFec, and OFec). The results are presented in Table 4 and are expressed in terms of EC_50_, units in mg/L.

In general, samples showed high antioxidant capacity. The Macedo sample exhibited the highest antioxidant capacity (EC_50_ values varied between 94 ± 2 and 326 ± 10 mg/L) and the Lousã sample the lowest (EC_50_ values varied between 332 ± 5 and 859 ± 6 mg/L) for the four methods studied. The MFec method gave the highest antioxidant capacity in all the samples, followed by the DPPH method. FRAP and OFec gave low antioxidant capacity values, with close values between the two. Regarding the antioxidant capacity of ascorbic acid, the DPPH and OFec methods gave lower and similar EC_50_ values, whereas the FRAP method gave a higher EC_50_ value. As expected, the propolis samples showed much higher values than the positive control, since it is a purified compound with high antioxidant capacity.

Two-way ANOVA without interaction was performed to test the differences between the propolis samples (factor 1, four samples) and their antioxidant capacity (factor 2, four methods). The ANOVA model was significant (*p*-value < 0.001), with an RSE of 7.1 and R^2^ of 0.9993, which means that the model explains 99.93% of the variability within the experimental data. Significant differences were found within each of the factors (*p*-value < 0.001). The overall data showed normality (Shapiro–Wilk test, with *p*-value = 0.292) and homogeneity of the values (Levene test, with *p*-value = 0.705).

The Macedo and Montesinho samples were statistically equal (mean EC_50_ values of 255 ± 138 mg/L and 280 ± 134 mg/L, respectively), while the Braga and Lousã samples are statistically different (mean EC_50_ values of 342 ± 195 mg/L and 618 ± 227 mg/L, respectively). Among the four methods tested to determine the antioxidant capacity of propolis samples, we found that the FRAP and OFec methods were statistically equivalent (mean EC_50_ values of 559 ± 153 mg/L and 507 ± 220 mg/L, respectively), while the DPPH and MFec methods were statistically different (mean EC_50_ values of 259 ± 139 mg/L and 170 ± 99 mg/L, respectively).

**Figure 3:**
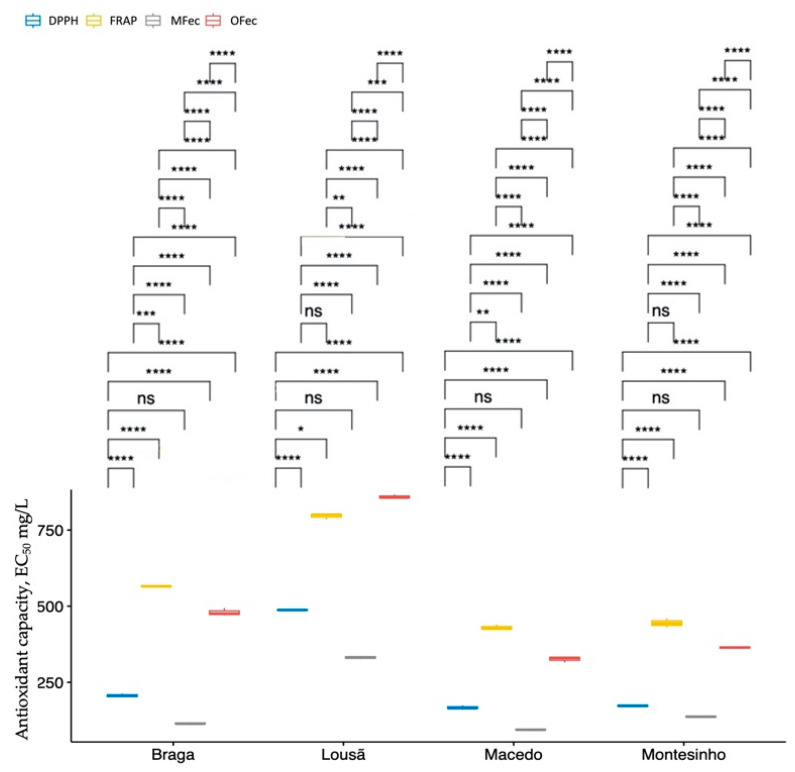
Box plots of each method of antioxidant capacity for each propolis sample. ns—not significant; the significance levels are translated by the following symbols: **** (*p*-value < 0.0001); *** (*p*-value < 0.001); ** (*p*-value < 0.01); * (*p*-value < 0.05); ns (*p*-value > 0.05).

To evaluate the antioxidant capacity of different propolis samples, box plots were created for each method employed. These box plots give an overview of the value distribution within each method, enabling simple comparison between samples. To further analyse the data, the means of each method were compared to identify any significant differences. These comparisons are displayed on the box plots, making it easy to identify which methods produced the greatest variation in antioxidant capacity between samples (Figure 3).

Figure 3 demonstrates the analytical methods that yielded the highest and lowest quantification of antioxidant capacity. The behaviour of the samples was generally comparable for all methods. In ascending order of EC50 values, the samples from Macedo, Montesinho, Braga, and finally Lousã showed the highest values. The samples from Macedo and Montesinho exhibited similar levels of antioxidant capacity, which was validated by ANOVA indicating that the samples were statistically equivalent.

#### 2.3.2. Correlation of Antioxidant Capacity with HBA, HCA, and FLAV

In evaluating the results of antioxidant capacity, we attempted to obtain estimation models related to the concentrations of HBA, HCA, and FLAV present in the propolis samples (as presented in the study by Paula et al. [31]).

The results of the estimation models obtained, as well as the values of RSE, R^2^, and *p*-value of method significance, are presented in Table 5.

Estimate models used:

log([antioxidant]) = b + a1 × log([HBA]) + a2 × log([HCA]) + a3 × log([FLA])(2)

From the analysis of Table 5, all the estimation models show an acceptable fit, but with marked differences. The presence of HBA and HCA compounds in propolis samples has an overall positive effect on the quantification of their antioxidant capacity. They only have a negative effect on the MFec method and had no significant effect on the DPPH method. The same cannot be said for flavonoids, as they tend to have a negative effect on antioxidant capacity in all methods except the MFec method, which showed no significant interaction.

**Figure 4:**
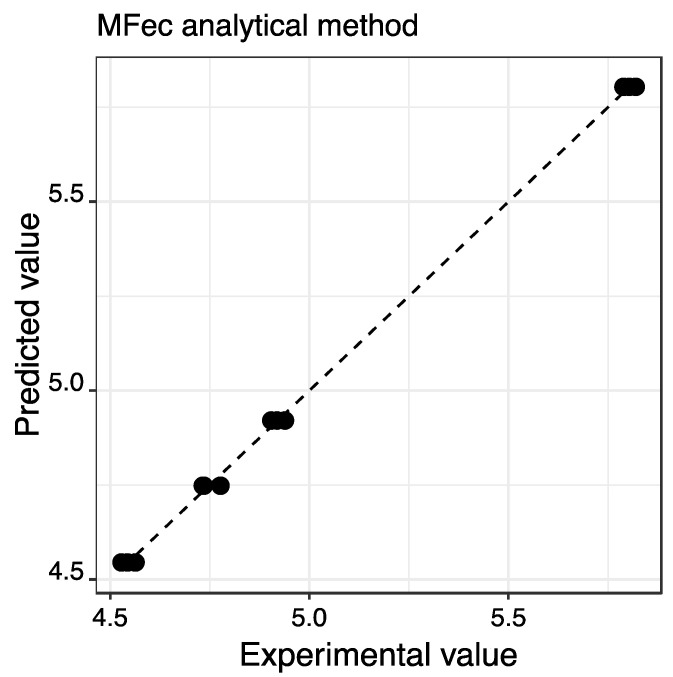
Linear relationship between the model estimation results and the MFec analysis method (EC_50_ mg/L).

Figure 4 shows the relationship between the data obtained by the model estimated in relation to the experimental values obtained with the MFec method, which shows an acceptable linear fit (significant estimation model, *p*-value < 0.001; slope = 0.999 ± 0.010 close to 1; intercept = 0.006 ± 0.055 close to 0; R^2^ = 0.999 close to 1), which is reflected in the low value of the RSE (0.019) and the acceptable coefficient of determination (0.9988) obtained in the estimation model.

### 2.4. UHPLC-DAD-ESI-MS^n^ Analysis

This study was carried out using UHPLC-DAD-ESI-MS^n^ in negative ion mode because of its greater sensitivity in analysing the different classes of polyphenols [32]. All the phytochemicals in propolis were characterised by their UV spectra (absorbance at 280 nm), retention time (*t*_R_), and MS/MS (MS^2^ and MS^3^) data and compared with the literature [33,34,35,36,37] (Supplementary Material).

Of a total of 62 compounds, 56 were identified, including 26 phenolic acids and their derivatives (caffeic acid, *p*-coumaric acid, isoferulic acid, ferulic acid, 3,4-dimethyl-caffeic acid, cinnamic acid, *p*-coumaric acid methyl ester, cinnamylidenacetic acid, caffeic acid isoprenyl ester, caffeic acid isoprenyl ester (isomer), caffeic acid phenylethyl ester, *p*-coumaric acid isoprenyl ester, *p*-coumaric benzyl ester, *p*-coumaric acid isoprenyl ester (isomer), caffeic acid cinnamyl ester (isomer), *p*-coumaric acid derivative, *p*-coumaric cinnamyl ester, caffeic acid derivative, *p*-coumaric acid-4-hydroxyphenylethyl ester dimer, caffeic acid cinnamyl ester (isomer), *p*-methoxy-cinnamic acid cinnamyl ester, *p*-methoxy-cinnamic acid cinnamyl ester (isomer)) and 30 flavonoids and derivatives (quercetin, pinobanksin-5-methyl-ether, quercetin-3-methyl-ether, apigenin, pinobanksin, kaempferol, isorhamnetin, pinocembrin-5-methyl-ether, kaempferol-methyl ether, quercetin-dimethyl-ether, galangin-5-methyl ether, pinobanksin-5-methyl-ether-3-*O*-acatate, rhamnetin, chrysin, acacetin, pinocembrin, galangin, kaempferide, pinobanksin-3-*O*-acetate, chrysoeriol-methyl-ether, pinocembrin-5-*O*-3-hydroxyl-4-methoxyphenylpropionate, pinobanksin-3-*O*-propionate, pinobanksin-5-methyl-ether-3-*O*-pentanoate, pinobanksin-7-methyl-ether-5-*O*-*p*-hydroxyphenylpropionate, pinobanksin-3-*O*-butyrate or isobutyrate, pinobanksin-3-*O*-penteonate, pinobanksin-3-*O*-pentenoate or 2-methylbutyrate, pinobanksin-*O*-hexenoate, pinobanksin-3-*O*-hexanoate).

Among them, quercetin-3-methyl-ether, acacetin, and chrysoriol-methyl-ether were only detected in the Lousã sample, while cinnamic acid, pinobankin-*O*-hexanoate, and pinobankin-3-*O*-hexanoate were absent in that sample.

The compounds with a larger peak area (although the concentration response can be different) in the four propolis samples were chrysin, caffeic acid isoprenyl ester, pinocembrin, galangin, pinobanksin-3-*O*-acetate, and caffeic acid phenylethyl ester.

**Figure 5:**
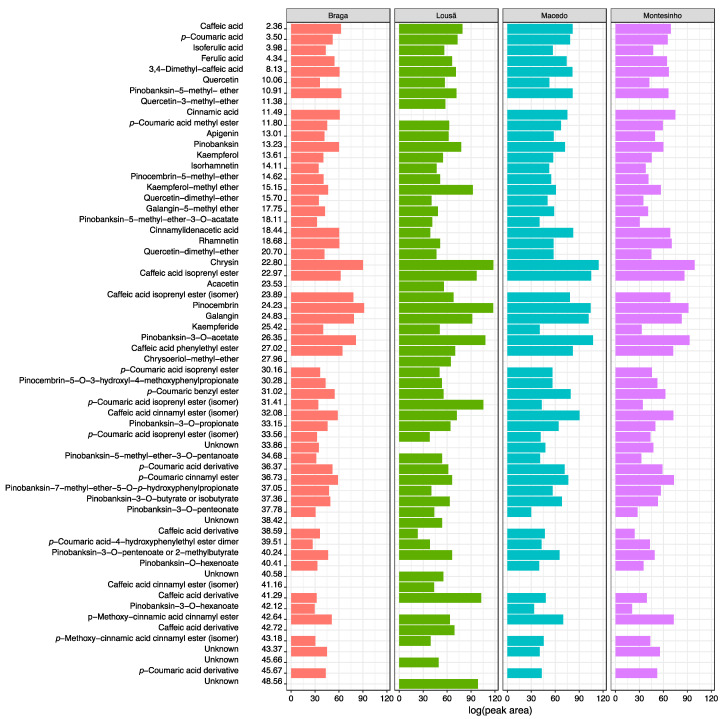
Profile of phenolic compounds obtained by UHPLC-DAD-ESI-MS^n^ in negative ion mode in the four propolis samples (retention times and peak areas of the chromatogram obtained by diode array detector, in logarithmic units).

Figure 5 shows the phenolic compounds present in the four propolis samples, their respective retention times, and the quantification of the areas of the chromatographic peaks in log units.

**Figure 6:**
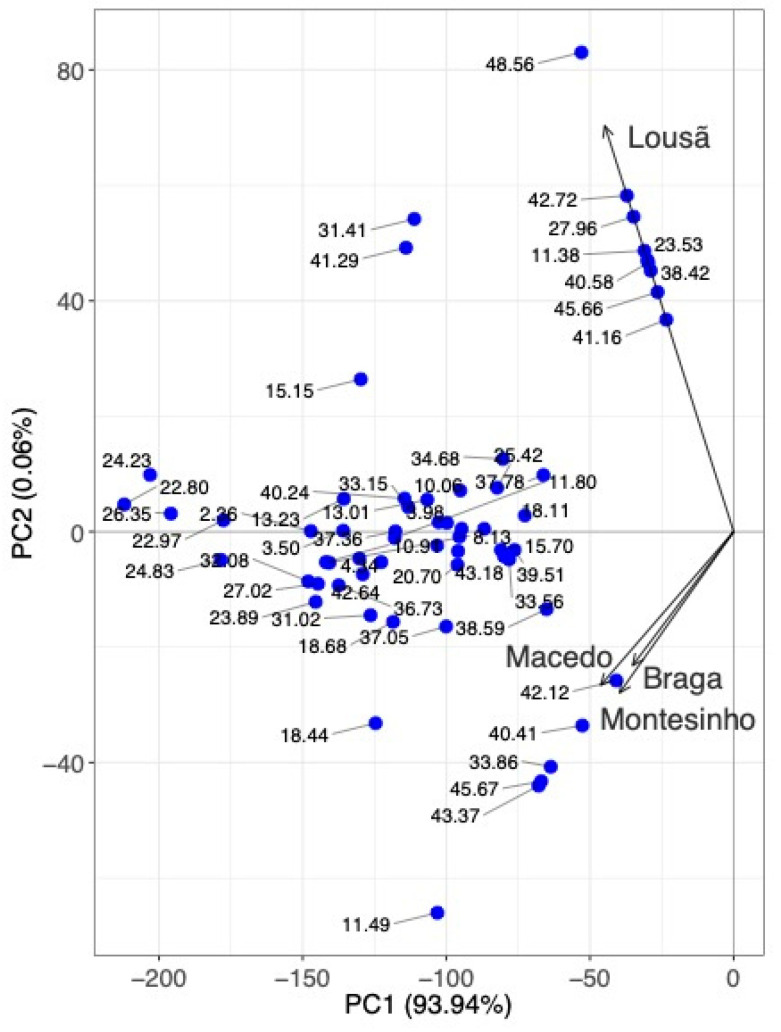
Biplot of the principal component analysis of the MS profile of phenolic compounds obtained for the four propolis samples.

A PCA was performed to analyse the mass spectrometry composition results and the UHPLC-DAD peak areas of each propolis sample. The Yeo–Johnson transformation was used to pre-process the data. With only four principal components, 100% of the variability in the data could be accounted for. Figure 6 shows the two-dimensional space represented by the first two principal components, which account for 99.67% of the variability (PC1: 93.94%; PC2: 5.73%).

PCA highlighted the differences between the samples, showing that the Macedo, Montesinho, and Braga samples were similar (correlation of 0.97), while the Lousã sample was significantly different (correlations of 0.30, 0.32, and 0.37 for the samples from Montesinho, Braga, and Macedo, respectively). The differences evidenced by PCA were high concentrations of the compounds caffeic acid derivative (rt. 40.58, 41.29, 42.72 min), caffeic acid cinnamyl ester (isomer) (rt. 41.16 min), chrysoeriol-methyl-ether (rt. 27.96 min), *p*-coumaric acid isoprenyl ester (isomer) (rt. 31.42 min), kaempferol-methyl ether (rt. 15.15 min), quercetin-3-methyl-ether (rt. 11.38 min), chrysin (rt. 22.80 min), acacetin (rt. 23.53 min), and unknown (rt. 38.42, 45.66, 48.56 min) and low concentration of the compounds cinnamic acid (rt. 11.49 min), cinnamylidenacetic acid (rt. 18.44 min), unknown (rt. 33.86, 43.37 min), pinobanksin-7-methyl-ether-5-*O*-*p*-hydroxyphenylpropionate (rt. 37.05 min), pinobanksin-*O*-hexenoate (rt. 40.41 min), pinobanksin-3-*O*-hexanoate (rt. 42.12 min), and *p*-coumaric acid derivative (rt. 45.67 min).

## 3. Discussion

### 3.1. Pollinic Analysis

In temperate regions of the world, *Populus* species have been described as the main source of propolis [38,39]. In this work, the four propolis samples have a high percentage of this pollen. This agrees with the works of Dias et al. [40], which analysed Portuguese propolis samples from different regions, and Falcão et al. [41], which analysed samples from temperate regions, including Portugal. It is stated that propolis derived from *Populus nigra* could be used in the pharmaceutical and/or food industry due to its rich phytochemical composition [42]. However, in this study, the sample with the highest *Populus* sp. content was not the one with the highest biological properties. Therefore, applications based only on pollen analysis are not a satisfactory approach to determine the bioactive properties of this natural product [43]. However, caution should be taken when relating the results to the propolis pollen profile as this may not be an accurate indicator of plant origin and could be dangerously misleading [44].

### 3.2. Quantification of Total Phenolic Compounds

Six different methods were used to quantify TPC: four spectrophotometric methods based on the F-C reagent and one based on spectra at 280 nm. The sixth method is an electrochemical method using square wave voltammetry. The differences obtained between the six methods for quantification of total phenolic compounds in propolis samples are discussed below.

The F-C method is based on electron transfer in which a mixture of two acids, phosphotungstic and phosphomolybdic, reduces phenols and produces a colour change measured at 765 nm [45,46]. This method is sensitive to pH, temperature, and reaction time [47,48]. The pH at the end of the reaction varies according to the method used, since the final concentrations of the reactants F-C and Na_2_CO_3_ differ between the methods. This explains the differences between the four F-C methods investigated in this work. According to the result of the ANOVA, the four F-C methods were grouped in pairs: F-C1 and F-C4 methods with high final concentrations of F-C (33 and 50%, respectively) and low concentrations of Na_2_CO_3_ (3.3 and 3.0%, respectively) and F-C2 and F-C3 methods with low concentrations of F-C reagent (5 and 2%, respectively) and Na_2_CO_3_ (3 and 0.8%, respectively). The methods using low concentrations of these reagents allowed higher quantification of TPC due to the lower pH at the end of the reaction. Lawag et al. [49] also concluded that lower pH values allowed higher quantification of TPC in honey samples. As with F-C methods, the spectrophotometric (SPECT) method is based on spectrophotometry and results can be expressed in simple terms as absorbance measured at 280 nm (A_280_) or converted to gallic acid equivalents [45,50]. The SPECT method is a faster and less expensive method for measuring the total phenolic content in samples. The SPECT method requires only one reagent, hydrochloric acid, which is readily available and much less expensive than the reagents used in the F-C method. In addition, SPECT requires no incubation time, making it faster than FC, which requires a long incubation period. The SPECT method gave a higher quantification of TPC compared to F-C and SWV methods. Way et al. [51] also found higher values for total phenolic compounds in cider samples using the spectrophotometric method (Somers) than the F-C method.

In recent years, electrochemical methods have been extensively investigated for the determination of phenolic compounds, mainly due to their simplicity, high sensitivity, rapid response, and low cost [52]. Most of the biological activity of these compounds is due to their ability to donate electrons to a wide range of receptor species. The redox potential of natural phenolic compounds covers a significant range, which is the first source of selection allowing the selective analysis of different electroactive compounds by different voltammetric techniques such as cyclic voltammetry (CV), differential pulse voltammetry (DPV), or square wave voltammetry (SWV) [53].

The SWV technique has several advantages over other voltammetric techniques such as CV and DPV, including the fact that it consumes fewer electroactive species, is faster, is more sensitive, takes much less time to analyse, and has fewer problems with electrode poisoning than other methods [54,55].

The TPC values obtained with the SWV method were lower than those obtained with the F-C and SPECT methods. As reported in previous publications [56,57], the values obtained with the F-C method are generally higher than those obtained with electrochemical analysis. This behaviour is attributed to the different oxidants used, i.e., the chemical reagents used in the F-C method and the potential applied for the oxidation reaction at the electrode surface in electrochemical sensors [58]. In the F-C method, the reagents oxidise not only the phenolic compounds but also other non-phenolic species that may be present in the sample. In the SWV method, only the phenolic compounds are oxidised by the oxidising potentials applied [58,59]. Regarding the correlation between TPC and the different groups of phenolic compounds, the results indicate that the presence of HBA and HCA compounds did not have a significant influence on the quantification of TPC in the majority of the methods tested. However, the HCA content present in the samples has a positive influence on the quantification of TPC in methods F-C2 and F-C3. These methods have higher TPC quantification. On the other hand, the presence of FLAV compounds in propolis samples can positively influence the quantification of TPC in all tested methods. Several works show that there is a positive correlation between TPC and flavonoid content [60,61]. However, no comparative work has been found relating TPC to HBA and HCA content. Therefore, to the authors’ knowledge, this is the first work that attempts to evaluate the influence of phenolic groups on the results obtained by different methods for total phenolic content. Overall, this study sheds light on the influence of different groups of phenolic compounds on TPC quantification and highlights the varying effectiveness of different methods in capturing total phenolic content. The results show that the F-C2 method had the strongest correlation with the three groups of phenolic compounds. Conversely, the results of the SPECT method, which measured higher levels of TPC in the samples, only correlated significantly with FLAV compounds, while the presence of HBA and HCA compounds had no significant effect.

### 3.3. Antioxidant Capacity

In this study, three different reducing power assays (FRAP, MFec, and OFec) and one radical scavenging assay (DPPH) were used to measure the antioxidant capacity of propolis samples from different geographical locations. The DPPH and FRAP methods are the most widely used assays to evaluate the antioxidant capacity of foods and biological extracts [47,62].

The principle of the DPPH method is based on the reaction between the free radical DPPH and antioxidants. The free radical DPPH has a stable deep purple colour. When DPPH radicals are allowed to react with antioxidants, the colour of the solution changes to yellow [47,63].

The FRAP, MFec, and OFec methods are based on the same principle. This is the reduction of ferric ions (Fe^3+^) to ferrous ions (Fe^2+^), forming a blue complex [64]. The three methods differ in the reagents used and the pH of the solution. The FRAP assay is a colorimetric method that exploits the ability of antioxidants to reduce the colourless [Fe^3+^-(2,4,6-tris(2-pirydyl)-s-triazine)2]^3+^ complex to the intense blue [Fe^2+^-(TPTZ)2]^2+^ complex in an acidic medium [65]. Such colour changes are measured spectrophotometrically at 593 nm. This method requires specific conditions, including an acidic medium (pH 3.6) to facilitate iron solubility. The low pH decreases the ionisation potential that drives electron transfer and increases the redox potential, causing a shift in the dominant reaction mechanism [66]. In the OFec method, a phosphate buffer at pH 6.6 was used and Prussian blue solution was measured at 700 nm. One of the differences between the OFec and MFec methods is that the modified method does not use pH 6.6 phosphate buffer. There is also no precipitation of the Prussian blue solution due to the stabilising effect of the sodium dodecyl sulphate (SDS) reagent [47,64]. Of the three iron-based methods, FRAP and MFec assays were carried out in acidic solution due to the hydrolysis of the ferric ion at a slightly neutral to acidic pH.

The results obtained by the four methods differed slightly. The MFec method allowed for quantifying higher antioxidant capacity in all the propolis samples, while the OFec and FRAP methods showed the lowest antioxidant capacity. It is natural that the results obtained from different antioxidant assays based on electron transfer give comparable but not identical results for antioxidants. This is due to the diversity of reaction conditions, such as redox potential, pH, and kinetics, of these assays.

Some authors [47,67,68] conclude that the FRAP method has limitations and needs to be modified. The fact that it is based on an aqueous solution (acetate buffer) limits the method to hydrophilic substances, whereas plant essential oils and their antioxidant components (i.e., terpenes) are hydrophobic. The same applies to the OFec method, which is also based on an acetate buffer. Ferricyanide methods can be a cheaper alternative to FRAP under certain conditions, with partially improved molar uptake (and thus sensitivity) of antioxidants, lower intercept values, wider linear range, and better additivity of total antioxidant capacity values of antioxidant components in mixtures.

Overall, the results showed a positive contribution of HBA and HCA content in determining antioxidant capacity. The FRAP and OFec methods showed the greatest contribution of these compounds to the determination of antioxidant capacity. However, the contribution of HCA was greater than that of HBA, which was confirmed by Natella et al. [69], who reported that HCA had stronger antioxidant capacity than HBA when the propenoic side chain was attached instead of the carboxyl group of benzoic acid derivatives. These two groups (HBA and HCA) showed antioxidant properties against different types of free radicals [70,71,72]. Velika et al. [73] investigated the relationship between HBA and antioxidant capacity and found that there was a positive correlation between them. They concluded that the structure and position of the hydroxyl group is very important for antioxidant capacity. Mazzone et al. [74] confirmed the antioxidant properties of HCA and derivatives. They indicated that a structural change in the ethylene spacer between the aromatic ring and the carboxyl functionality increases the antioxidant capacity of the derivatives. The antioxidant capacity of phenolic acids is based on the phenolic hydroxyl, so the number and position of phenolic hydroxyls are directly related to their antioxidant capacity [75,76]. In addition, the methoxy and carboxylic acid groups also have important effects on the antioxidant capacity of phenolic acids [76,77]. This may be the reason for the results obtained regarding the contributions of HBA and HCA in the different methods for the determination of antioxidant capacity.

The correlation of antioxidant capacity with FLAV shows that flavonoids contribute negatively to the determination of antioxidant capacity in all tested methods, except in the MFec method. The antioxidant capacity of flavonoids is mainly related to their chemical structure and, as with phenolic acids, is based on the O-H bond dissociation energy value [76,78]. Some authors have also found a negative correlation between flavonoids and antioxidant capacity in propolis extract [10,60,79]. Khiya et al. [80] also found a negative correlation between flavonoids and the DPPH and FRAP methods in leaf extracts of *Salvia officinalis*. The results suggest that the OFec and FRAP methods are most closely related to the three groups of phenolic compounds but did not reveal a higher antioxidant capacity in propolis samples. This suggests that the negative influence of flavonoids may interfere with the determination of the antioxidant capacity of the samples, since the MFec method showed the highest antioxidant capacity.

The effectiveness of antioxidants depends on several factors, the most important of which are structural properties, temperature, properties of the substrate susceptible to oxidation, concentration, the presence of synergistic and pro-oxidant compounds, and the physical state of the system [76]. It should be noted that the methods used to determine antioxidant capacity have different reaction systems. The DPPH assay reacts in an ethanol system whereas the FRAP, OFec, and MFec methods react in a water system. It should also be noted that flavonoids can act as antioxidants by different mechanisms such as hydrogen atom transfer, single electron transfer, and transition metal chelation [76]. Kiokas et al. [81] stated that the strong antioxidant and biochemical potential of these natural products may be linked to the synergistic effect of their individual phenolic compounds. However, antagonistic effects cannot be neglected either. The study highlights the positive correlation between total phenolic compound content and antioxidant capacity, as reported by several authors [60,82,83]. The sample with a higher TPC content also showed higher antioxidant capacity.

### 3.4. UHPLC-DAD-ESI-MS^n^ Analysis

The UHPLC-DAD-ESI-MS/MS method was used for the separation and identification by analytical chromatography of 56 compounds from propolis extracts. Characteristic peaks were identified by comparing their chromatographic behaviour, UV spectra, and MS information with those of reference compounds, referring to previous studies [33,34,35,36,37,84].

Only the phenolic compounds HCA and FLAV were detected and identified in the propolis samples. Falcão et al. [35] also identified only these groups of compounds in propolis samples. In the present study, flavonoids identified as chrysin, pinocembrin, galangin, and pi-nobanksin-3-*O*-acetate were detected in the four propolis extracts with large peak areas, thus suggesting their predominance. Falcão et al. [41] have also identified these four flavonoids as the main compounds in Portuguese propolis extracts. Consistent with this, Yuan et al. [84] and Avula et al. [33] identified these four flavonoids as the most abundant in propolis samples using three extraction methods and in propolis extracts from different geographical locations, respectively. Guzelmeric et al. [43] have also identified pinocembrin, galangin, and chrysin in propolis extracts. In addition, Bhuyan et al. [85] report pinocembrin and galangin as significant components in two propolis samples from Australia.

As for phenolic acids, the largest peak areas were found for caffeic acid derivatives such as isoprenyl ester and phenylethyl ester. These data also agree with literature data, as caffeic acid derivatives were identified as the main components of the *O*-subtype of Serbian and Turkish propolis samples [43,86].

Globally, the presence of these compounds (phenolic acids and flavonoids) in propolis samples is characteristic of poplar buds [41,87,87]. This agrees with the analysis of the pollen, which showed that the pollen of the species *Populus* sp. was present in all samples.

## 4. Materials and Methods

### 4.1. Chemicals and Reagents

All reagents were of analytical quality and used as purchased. The standard used was gallic acid (1-Hidrate) from Panreac (99%, Barcelona, Spain). As solvents, absolute ethanol (EtOH) was acquired from Panreac (HPLC quality, Spain, 99.9%); diethyl ether from Carlo Erba. Other reagents were hydrochloric acid (HCl) from Carlo Erba (Val-de-Reuil, France, 37% and d = 1,18); glacial acetic acid and acetic anhydride from Merck (Darmstadt, Germany); sulphuric acid (H_2_SO_4_) from José Manuel Gomes dos Santos; glycerol from Analar Normapur (VWR Chemicals); potassium hydroxide (KOH), sodium carbonate (Na_2_CO_3_), sodium chloride (NaCl), sodium hydrogen phosphate (Na_2_HPO_4_), and potassium dihydrogen phosphate (KH_2_PO_4_) were acquired from Panreac (Spain); Folin–Ciocalteu reagent and potassium chloride (KCl) were acquired from Scharlau (Spain); potassium hexacyanoferrate(III) (K_3_Fe(CN)_6_), 2,3,5-Triphenyltetrazolium chloride (TPTZ), and ferric chloride hexahydrate (FeCl_3_·6H_2_O) were acquired from ACRÖS Organics (Geel, Belgium); potassium hexacyanoferrate(II) trihydrate (K_4_Fe(CN)_6_·3H_2_O) were from Riedel-de-Haën (Hanover, German); sodium dodecyl sulphate (SDS), sodium acetic acid trihydrate salt (CH_3_COOH·3H_2_O), and 2,2-diphenyl-1-picrylhydrazyl (DPPH) were from Sigma Aldrich (Darmstadt, Germany); and trichloroacetic acid (TCA) was from Biochem Chemopharma (Cosne-Cours-sur-Loire, France). The deionised water used in all analytical work was of type II.

### 4.2. Propolis Samples

Propolis samples were collected in three locations in the northern region of Portugal (Montesinho, in the Trás-os-Montes sub-region; Macedo de Cavaleiros, in the Trás-os-Montes sub-region; Braga, Minho sub-region) and one in the central region (Lousã, Baixo Mondego). Samples were prepared by mixing 5 g of raw propolis with absolute ethanol (1:5, *w*/*v*) and left overnight with stirring (60 rpm). After this step, the solution obtained was filtered (Whatman n 4 filter paper). Two further ethanolic extractions were carried out using the same procedures. The combined ethanolic extracts were stored at low temperatures (−18 °C) and filtered after 12 h to remove wax; this procedure was repeated two more times. The ethanol was evaporated with a rotary evaporator (IKA model RV8). To the propolis extract obtained, 100 mL of diethyl ether and 100 mL of deionised water were added to obtain two visible phases. The sample was treated with diethyl ether solvent to extract as many phenolic compounds as possible. The supernatant (diethyl ether) was then transferred to a new beaker, and the solvent extraction process was repeated three more times until a transparent area was obtained between the two visible phases. From the resulting extracts (approximate yield of 2 g of purified propolis extract), 0.1 g of extract from each sample was weighed and dissolved in 25 mL of 80% absolute ethanol. This propolis solution was used to determine the total phenolic content using a gallic acid standard calibration line. To determine the antioxidant capacity, different concentrations of propolis (from 12–1200 mg/L) were prepared to calculate the EC_50_ value in mg/L.

### 4.3. Pollinic Analysis

Pollen analysis was performed according to the method described by Barth et al. [88]. The pollen collection, within 0.5 g of propolis, was mixed with 15 mL of ethanol for 24 h. After 24 h, the preparation was centrifuged for 15 min at 2200 rpm (Eppendorf centrifuge 5810 R, Hamburg, Germany). To the sediment obtained by centrifugation, 3 mL of 10% (*w*/*v*) KOH was added and it was placed in a water bath and boiled for 2 min. The mixture was centrifuged at 2200 rpm for 10 min and, after washing with deionised water, centrifuged again at 2200 rpm for 10 min. The sediment was left overnight in 5 mL glacial acetic acid. This mixture was centrifuged at 2000 rpm for 17 min. Then, 5 mL of the mixture of acetic anhydride and sulphuric acid (9:1 *v*/*v*) was added and heated in a water bath at 80 °C for 3 min. The mixture was centrifuged at 2000 rpm for 17 min. The resulting sediment was washed with deionised water, centrifuged at 2200 rpm for 10 min, then washed with 50% glycerol–water and centrifuged at 2000 rpm for a further 15 min. The sediment was mounted on glycerol–gelatine.

### 4.4. Quantification of Phenolic Compounds

Determination of total phenolic content (TPC) in the ethanolic extract of the different propolis was carried out spectrophotometrically (estimated by a colorimetric assay based on four different procedures using Folin–Ciocalteu reagent (F-C reagent) and spectrophotometric method). Phenolic compounds were also determined electrochemically (cyclic voltammetry method). The absorbance was measured in a PC VWR UV–Vis spectrophotometer and the results were expressed as mg of gallic acid equivalents/g of extract.

- F-C1 assay: described by Moreira et al. [89]. The reaction of 0.5 mL propolis extract mixed with 0.5 mL of the F-C reagent and 0.5 mL of 10% sodium carbonate (Na_2_CO_3_) was kept in the dark at room temperature for 60 min (final concentrations of F-C and Na_2_CO_3_ in solution, 33% and 3%, respectively), after which the absorbance was read at 700 nm.
- F-C2 assay: described by Obied et al. [90]. Propolis extract (0.1 mL) was added to a 10 mL volumetric flask containing 7 mL water. Then, 0.5 mL of F-C reagent was added and, after 1 min, 1.5 mL of Na_2_CO_3_ (20% *w*/*v*) was added (final concentrations of F-C and Na_2_CO_3_ in solution, 5% and 3%, respectively). The flask was shaken, and the volume was made up to 10 mL with water. The flask was kept for 60 min in the dark at room temperature. The absorbance was read at 760 nm.
- F-C3 assay: described by Shaghaghi et al. [91]. Aliquots of 0.5 mL of samples were mixed with 2.4 mL of deionised water, 2 mL of 2% Na_2_CO_3_, and 0.1 mL of F-C reagent (final concentrations of F-C and Na_2_CO_3_ in solution, 2% and 0.8%, respectively). After incubation at room temperature for 60 min, the absorbance of the reaction mixture was measured at 750 nm.
- F-C4 assay: described by Metrouh-Amir et al. [92]. First, 0.2 mL of sample extract was mixed with 1 mL of F-C reagent and 0.8 mL of 7.5% (*w*/*v*) Na_2_CO_3_ was added (final concentrations of F-C and Na_2_CO_3_ in solution, 50% and 3%, respectively). After incubation for 60 min at room temperature in the dark, the absorbance was measured at 740 nm.
- Spectrophotometry (SPECT): described by Obied et al. [90]. Aqueous ethanol (95% *v*/*v*; 1 mL) containing 0.1% hydrochloric acid was added to the dilute extract (1 mL) in a 10 mL volumetric flask, and the volume was made up to 10 mL with 2% hydrochloric acid. The absorbance was measured at 280 nm to determine total biophenols using gallic acid as standard.
- Square wave voltammetry (SWV): described by Meirinho et al. [93] with some modifications. Phosphate-buffered saline (PBS) was prepared to contain 137 mM NaCl, 2.7 mM KCl, 8.1 mM Na_2_HPO_4_, and 1.47 mM KH_2_PO_4_, with pH adjusted to 7.4. The redox probe was always freshly prepared in order to obtain a solution with concentration of 5 mM of K_3_Fe(CN)_6_ and K_4_Fe(CN)_6_ (1:1) and 10 mM of KCl in 100 mL of PBS, at pH 7.4. Square wave voltammetry (SWV) at a potential range of −0.2 to 1.1 V was used to evaluate the reducing properties of the oxides. The amplitude was set at 100 mV and the frequency at 50 Hz. At the additive level, the step size was set to 5 mV. Platinum electrodes from Micrux Technologies were used for this analysis.

### 4.5. Antioxidant Capacity

The concentration of propolis corresponding to 0.5 of the absorbance (EC_50_) was calculated from a linear regression analysis of absorbances as a function of extract concentrations in solution using Excel (Microsoft Corporation, Redmond, WA, USA). The EC_50_ results for the four methods were expressed in mg/L.

#### 4.5.1. 2,2-Diphenyl-1-picrylhydrazyl (DPPH): Described by Hatano et al. [94]

An aliquot of 0.3 mL of propolis extract was mixed with 2.7 mL of DPPH reagent (2.0 × 10^−4^ M). The mixture was allowed to stand in the dark for 60 min. The absorbance of the solutions was measured at 517 nm. The inhibitory effect of DPPH was calculated from the percentage of DPPH discoloration using the following equation:

% inhibition = [(ADPPH − AS)/ADPPH] × 100(3)

where AS is the absorbance of the solution when the sample extract was added and ADPPH is the absorbance of the DPPH solution. The concentration of extract giving 50% inhibition (EC_50_ mg/L) was calculated from the graph of the effect of percentage of removal as a function of the concentration of extract in the solution.

#### 4.5.2. Ferric-Reducing Antioxidant Power Method (FRAP): Described by Berker et al. [64]

The FRAP reagent was prepared using a buffer solution of 0.3 M sodium acetic acid trihydrate salt (CH_3_COOH·3H_2_O) at pH 3.6 to which glacial acetic acid, a solution of TPTZ dissolved in 96% EtOH (1.0 × 10^−2^ M), and FeCl_3_·6H_2_O solution (2.0 × 10^−2^ M) were added in a volume ratio of 10:1:1. The FRAP reagent was prepared and used fresh. Then, 0.1 mL of sample was mixed with 3 mL of FRAP reagent and 0.3 mL of deionised water. After 6 min the absorbance was read at 595 nm.

#### 4.5.3. Original Ferricyanide Method (OFec): Described by Berker et al. [64]

A mixture of 1.0 mL sample, 2.5 mL 0.2 M phosphate buffer (pH 6.6), and 2.5 mL K_3_Fe(CN)_6_ solution (1%) was incubated for 20 min at 50 °C in a water bath. The incubated mixture was allowed to cool to room temperature and 2.5 mL of TCA (10%) was added. The solution was thoroughly mixed, an aliquot of 2.5 mL was taken, and 2.5 mL of water followed by 0.5 mL of FeCl_3_-6H_2_O solution (0.1%) was added. Absorbance was measured at 700 nm after 2 min.

#### 4.5.4. Modified Ferricyanide Method (MFec): Described by Berker et al. [64]

The mixture of 1.0 mL of propolis extract, 5 mL of deionised water, 1.5 mL of HCl (1 M), 1.5 mL of ferricyanide solution (1%), 0.5 mL of SDS (1%), and 0.5 mL of FeCl_3_·6H_2_O (0.2%) was incubated at 50 °C on a water bath for 20 min. After that, it was left to cool to room temperature and the absorbance was measured at 750 nm.

### 4.6. Quantification of HBA, HCA, and FLAV

The quantification of the levels of HBA, HCA, and FLAV present in the propolis samples was carried out according to the method of Obied et al. [90], and the results cited in this paper were published in the work of Paula et al. [31]. The simultaneous quantification of the three classes of phenolic compounds (HBA, HCA, and FLAV) was carried out using multivariate calibrations obtained with mixed standard solutions of gallic acid, ferulic acid, and quercetin (representative compounds of these classes) and the corresponding UV–Vis spectra.

### 4.7. Compound Identification by UHPLC-DAD-ESI-MS^n^

The UHPLC-DAD-ESI-MS^n^ analyses were performed on a Finnigan Surveyor Plus HPLC instrument equipped with a DAD and coupled to an MS. The chromatographic system consisted of a quaternary pump, an autosampler, a degasser, a photodiode array detector, and an automatic thermostatic column compartment. The HPLC was run on a Macherey-Nagel Nucleosil C18 column (250 mm_4 mm i.d.; 5 mm particle diameter, end-capped) and the temperature was maintained at 25 °C. The mobile phase consisted of (A) 0.1% (*v*/*v*) formic acid in water and (B) acetonitrile, previously degassed and filtered. The solvent gradient started with 80% A and 20% B, reached 30% B at 10 min, 40% B at 40 min, 60% B at 60 min, 90% B at 80 min, and returned to the initial conditions. For the HPLC analysis, the propolis extract (0.1 g) was dissolved in 25 mL of 80% ethanol. All samples were filtered through a 0.2 mm nylon membrane (Whatman). The flow rate was 1 mL/min, and 200 mL/min was injected into the MS. Spectral data were collected for all peaks in the 200–600 nm range. The MS used was a Finnigan Surveyor LCQ XP MAX quadrupole ion trap MS equipped with an ESI source. The Thermo Xcalibur Roadmap data system was used for control and data acquisition. Nitrogen of over 99% purity was used and the gas pressure was 520 kPa (75 psi). The instrument was operated in negative ion mode with the ESI needle voltage set at 5.00 kV and the ESI capillary temperature set at 325 °C. The full scan covered the mass range from *m*/*z* 50 to 1000. MS^n^ data were acquired simultaneously for the selected precursor ion. The collision-induced decomposition (CID)-MS-MS and MS^n^ experiments were performed using helium as the collision gas with a collision energy of 25–40 eV. The quantification of the areas was carried out and the data for the identification of the phenolic compounds were obtained using the Xcalibur 2.2 software of the Thermo Scientific™ LC-MS systems, which allows data acquisition and processing. The identification of phenolic compounds was based on the interpretation of ultraviolet (UV) spectrophotometry and mass spectrometry (MS and MS/MS) data and comparison with the literature [33,34,35,36,37].

### 4.8. Statistical Analysis

All assays were performed in triplicate and results are presented as mean ± standard deviation. All statistical analyses were performed using R software (R version 3.3.2, 31 October 2016), a free software environment for statistical computing and graphics. Two-factor ANOVA without interaction was used to evaluate two independent variables (factors) that influence the dependent variable. It was applied to verify the differences between propolis samples (4 samples) and TPC quantification (6 methods), as well as to test the differences between propolis samples (4 samples) and their antioxidant capacity (4 methods). Multiple linear regression models were established between TPC or antioxidant capacity values and HBA, HCA, and FLAV contents. The intercept was removed if it was not significant (*p*-value > 0.05). The results are considered satisfactory when the linear regression parameters are close to the theoretical values [95,96]: ‘zero’ (0) for root-square error (RSE) and intercept; ‘one’ (1) for slope and coefficient of determination (R^2^). Principal component analysis was applied to evaluate the MS data, to understand the variability of the propolis samples, and to define which phenolic compounds contribute to their distinctiveness. PCA (using R software) was performed on the covariance matrix, ignoring the center and scale transformation, with only the pre-treatment with the Yeo–Johnson transformation.

## 5. Conclusions

The pollinic analysis of the propolis samples showed a similar pollen profile, which is in agreement with the results of the GC-MS/MS analysis.

The results of TPC and antioxidant capacity demonstrated the importance of carefully selecting the method for determining these properties in propolis samples, as different methods can yield different results. Based on the linear relationship between the content of HBA, HCA, and FLAV and the results of TPC and antioxidant properties, it was found that the presence of HBA and HCA compounds in propolis samples can have a positive effect on the quantification of TPC and antioxidant capacity, while flavonoids can have a positive effect on the quantification of TPC and a negative effect on antioxidant capacity in most of the methods. The methods that gave the highest values for TPC (SPECT) and antioxidant capacity (MFec) did not correspond to the methods that showed the highest correlation with the three groups of phenolic compounds (F-C2, OFec, and FRAP). This is due to the fact that the methods correlate differently with the HBA, HCA, and FLAV compounds present in the samples. Therefore, the choice of method depends on the aim of the work and the sample to be analysed. It is important to note that the bioactive effects of propolis are still an active area of research and the specific mechanisms and contributions of each component are not yet fully understood. However, it is widely accepted that the bioactive properties of propolis result from the collective action of its various constituents, including, but not limited to, phenolic compounds.

Using the UHPLC-DAD-ESI-MS^n^ technique, most of the compounds present in the propolis samples were identified. The study revealed significant compositional differences between the Lousã sample and the other samples, as well as the variability in chemical composition of propolis samples from different geographical origins. It is noteworthy that the only phenolic acids detected in the four propolis samples were HCAs and their derivatives, which could be related to the fact that only the negative ion mode was used.

To evaluate the potentiality of propolis samples in human health, it is crucial to have analytical data about composition (qualitative and quantitative) and biological properties, as these results are related to the overall synergetic and antagonistic effects. Therefore, research in this area needs to be intensified to develop methods related to each group of phenolic compounds. It is also necessary to identify the remaining groups of compounds (e.g., terpenes) that may interfere with the biological properties of this natural product.

Overall, this study provides valuable insights into the composition and properties of propolis samples from Portugal, which can be useful in developing natural products and supplements and also in studying the performance of different analytical methodologies.

## Figures and Tables

**Table 1:** Pollen profile of the propolis samples (% values).

Samples	Braga	Lousã	Macedo	Montesinho
*Populus* sp.	21.4 ± 1.9	51.5 ± 1.5	20.2 ± 0.2	27.4 ± 1.6
*Trifolium repens*	25.6 ± 0.1			
*Cistus ladanifer*	19.9 ± 1.5		2.80 ± 0.6	
*Quercus* sp.			19.9 ± 2.4	5.50 ± 0.6
*Pinus nigra*	18.7 ± 0.5			10.1 ± 0.3
*Leontodon* sp.		5.50 ± 0.5	11.4 ± 2.5	3.70 ± 0.4
*Castanea sativa*		11.0 ± 1.0	7.0 ± 0.4	35.0 ± 2.0
*Euphorbia* sp.		3.50 ± 0.5	10.9 ± 0.5	
*Echium vulgare*	1.95 ± 0.15	5.50 ± 0.5	3.55 ± 0.85	4.35 ± 0.55
*Olea europacea*			6.70 ± 0.9	7.55 ± 0.65

**Table 2:** Total phenolic compounds (mg GAE/g of extract) determined using six different methods.

Samples	F-C1 ^c^	F-C2 ^b^	F-C3 ^b^	F-C4 ^c^	SPECT ^a^	SWV ^d^
Braga ^b^	146 ± 4	220 ± 7	255 ± 6	126 ± 3	460 ± 8	46 ± 11
Lousã ^c^	79 ± 2	117 ± 2	105 ± 6	75 ± 2	263 ± 6	43 ± 1
Macedo ^a^	168 ± 3	258 ± 6	289 ± 9	156 ± 7	503 ± 2	49 ± 3
Montesinho ^b^	147 ± 5	221 ± 4	235 ± 7	139 ± 27	465 ± 16	60 ± 6

F-C1 to F-C4, Folin–Ciocalteu colorimetric method; SPECT, spectrophotometric method; SWV, square wave voltammetry method; different letters indicate significant differences.

**Table 3:** Relationship between the results of total phenolics obtained by 6 different methods and the total concentrations of HBA, HCA, and FLAV present in propolis samples.

Method	RSE	R^2^	*p*-Value	b ± s	HBA ± s	HCA ± s	FLAV ± s
FC1	0.032	0.9903	<0.001	−11.3 ± 0.5	ns	ns	3.1 ± 0.1
FC2	0.024	0.9960	<0.001	−10.3 ± 1.4	−0.5 ± 0.1	0.7 ± 0.2	2.7 ± 0.5
FC3	0.039	0.9920	<0.001	−5.8 ± 0.3	ns	2.0 ± 0.1	ns
FC4	0.106	0.8907	<0.001	−10.5 ± 1.7	ns	ns	2.9 ± 0.3
SPECT	0.021	0.9944	<0.001	−8.4 ± 0.3	ns	ns	2.7 ± 0.1
SWV					ns	ns	ns

ns—not significant.

**Table 4:** Antioxidant capacity (EC_50_, mg/L) was determined using four different methods.

Samples	DPPH ^b^	FRAP ^a^	MFec ^c^	OFec ^a^
Braga ^b^	206 ± 6	566 ± 1	115 ± 3	480±12
Lousã ^a^	488 ± 2	795 ± 10	332 ± 5	859 ± 6
Macedo ^c^	168 ± 5	430 ± 8	94 ± 2	326 ± 10
Montesinho ^c^	173 ± 3	445 ± 15	137 ± 2	364 ± 3
L-ascorbic acid	23 ± 0.5	71 ± 0.4	42 ± 3	22 ± 0.1

DPPH, 2,2-diphenyl-1-picrylhydrazyl; FRAP, ferric-reducing antioxidant power; MFec, modified ferricyanide method; OFec, original ferricyanide method; different letters indicate significant differences.

**Table 5:** Correlation between the antioxidant capacity obtained by 4 different methods and the concentrations of HBA, HCA, and FLAV present in the propolis samples.

Method	RSE	R^2^	*p*-Value	b ± s	HBA ± s	HCA ± s	FLAV ± s
DPPH	0.022	0.9981	<0.001	38.1 ± 1.2	ns	1.6 ± 0.2	−7.9 ± 0.5
OFec	0.021	0.9979	<0.001	43.2 ± 1.2	1.3 ± 0.1	1.9 ± 0.2	−10.3 ± 0.5
MFec	0.019	0.9988	<0.001	18.3 ± 0.2	0.9 ± 0.1	−3.2 ± 0.1	ns
FRAP	0.020	0.9956	<0.001	34.3 ± 1.2	0.9 ± 0.1	1.9 ± 0.2	−8.3 ± 0.4

ns—not significant.

## Data Availability

Data available on request.

## Supplementary Materials

The following supporting information can be downloaded at: https://www.mdpi.com/article/10.3390/molecules28124847/s1, Table S1. Characterisation of phenolic compounds from Portuguese propolis by UHPLC-DAD-ESI-MSn. Figure S1. Chromatograms with UV detector (280 nm) of the four propolis samples.

## Sample Availability

Samples of the compounds are available from the authors.
