# Supplementary material for: Comparative Methods to Evaluate the Antioxidant Capacity of Propolis: An Attempt to Explain the Differences
Source: Molecules. 2023 Jun 19;28(12):4847. doi: 10.3390/molecules28124847 (PMC10303542; doi:10.3390/molecules28124847)
Supplement: Supplementary file 1 [file molecules-28-04847-s001.zip › molecules-2401641-supplementary.pdf]

Table S1. Characterisation of phenolic compounds from Portuguese propolis by UHPLC-DAD-ESI-MSn.

Nr	t_R (min)	UV max	$[M-H]^-$ m/z	UHPLC/ESI-MS m/z (% base peak)	Compound
**1**	2.36	297, 323	179	MS^2[179]: 135 (100)	Caffeic acid
**2**	3.50	309	163	MS^2 [163]: 119 (100)	*p*-Coumaric acid
**3**	3.98	295, 320	193	MS^2 [193]: 178 (56), 149 (100), 134 (25)	Isoferulic acid
**4**	4.35	299, 322	193	MS^2[193]: 178 (100), 149 (8), 134 (15)	Ferulic acid
**5**	8.13	295, 321	207	MS^2 [207]: 163 (100), 133 (80), 131 (60), 103 (100)	3,4-Dimethyl-caffeic acid
**6**	10.06	255, 368	301	MS^2 [301]: 179 (100), 151 (46)	Quercetin
**7**	10.91	287	285	MS^2[285]: 267 (100), 252 (8), 239 (40)	Pinobanksin-5-methyl- ether
**8**	11.38	257, 355	315	MS^2[315]: 300 (100); MS^3[300]: 271 (100), 255 (56), 151 (<1)	Quercetin-3-methyl-ether
**9**	11.49	275	147	MS^2[147]: 103 (100)	Cinnamic acid
**10**	11.80	308	177, $[M+46]^-$:223	MS^2[177]:	*p*-Coumaric acid methyl ester
**11**	13.01	267, 338	269	MS^2[269]: 225 (100), 201 (28), 151 (20), 149 (40)	Apigenin
**12**	13.23	291	271	MS^2[271]: 253 (100), 225 (28)	Pinobanksin
**13**	13.61	265, 367	285	MS^2[285]: 285 (100), 257 (43), 213 (40), 151 (47)	Kaempferol
**14**	14.11	255, 368	315	MS^2[315]: 300 (100); MS^3[300]: 283 (34), 271 (100), 227 (39), 151 (90)	Isorhamnetin
**15**	14.62	286	269	MS^2[269]: 254 (100), 227 (100), 165 (44)	Pinocembrin-5-methyl-ether
**16**	15.15	266, 351	299	MS^2[299]: 284 (100)	Kaempferol-methyl ether
**17**	15.70	255, 355	329	MS^2[329]: 314 (100); MS^3[314]: 299 (100), 285 (50), 271 (43), 243 (10)	Quercetin-dimethyl-ether
**18**	17.75	261, 300sh, 352	283	MS^2[283]: 268 (100), 240 (12), 239 (25)	Galangin-5-methyl ether
**19**	18.11	290	327	MS^2[327]: 285 (100), 267 (7), 239 (7)	Pinobanksin-5-methyl-ether-3-*O*-acetate
**20**	18.44	311	173	MS^2[173]: 129 (100), 102 (2)	Cinnamylidenacetic acid
**21**	18.68	256, 367	315	MS^2[315]: 300 (40), 287 (19), 193 (45), 165 (100)	Rhamnetin
**22**	20.70	255, 355	329	MS^2[329]: 314 (100); MS^3[314]: 299 (100), 271 (10)	Quercetin-dimethyl ether
**23**	22.80	268, 315	253	MS^2[253]: 253 (86), 209 (100), 180 (15), 165 (10)	Chrysin
**24**	22.97	297, 324	247	MS^2[247]: 179 (100), 135 (14)	Caffeic acid isoprenyl ester
**25**	23.53	268, 331	283	MS^2[283]: 268 (100)	Acacetin
**26**	23.89	299, 325	247	MS^2[247]: 179 (100), 135 (14)	Caffeic acid isoprenyl ester (isomer)

Table S1 (continued). Characterisation of phenolic compounds from Portuguese propolis by UHPLC-DAD-ESI-MSn.

Nr	t_R (min)	UV max	[M-H]$^-$ m/z	UHPLC/ESI-MS m/z (% base peak)	Compound
**27**	24.23	290	255	MS^2[255]: 213 (58), 211 (24), 151 (12)	Pinocembrin
**28**	24.83	266, 290sh,358	269	MS^2[269]: 269 (100), 241 (<1), 227 (<1), 213 (<1), 197 (<1)	Galangin
**29**	25.42	266, 363	299	MS^2[299]:284 (100), 271 (30), 255 (10), 240 (10), 165 (47)	Kaempferide
**30**	26.35	293	313	MS^2[313]: 271 (18), 253 (100)	Pinobanksin-3-*O*-acetate
**31**	27.02	300, 325	283	MS^2[283]: 179 (100), 135 (20)	Caffeic acid phenylethyl ester
**32**	27.96	267, 345	313	MS^2[313]: 298 (100); MS^3[298]: 283 (100), 269 (<1), 255 (5)	Chrysoeriol-methyl-ether
**33**	30.16	310	231	MS^2[231]: 163 (100), 119 (14)	*p*-Coumaric acid isoprenyl ester
**34**	30.28	295, 313	433	MS^2[433]: 415 (4), 401 (32), 309 (100), 323 (5), 294 (7), 269 (7)	Pinocembrin-5-*O*-3-hydroxyl-4-methoxyphenylpropionate
**35**	31.02	313	253	MS^2[253]: 209 (20), 163 (100), 145 (10), 118 (65)	*p*-Coumaric benzyl ester
**36**	31.42	313	231	MS^2[231]: 163 (100), 119 (12)	*p*-Coumaric acid isoprenyl ester (isomer)
**37**	32.08	294, 325	295	MS^2[295]: 251 (66), 211 (49), 178 (100), 134 (66)	Caffeic acid cinnamyl ester (isomer)
**38**	33.15	293	327	MS^2[327]: 271 (10), 253 (100)	Pinobanksin-3-*O*-propionate
**39**	33.56	313	231	MS^2[231]: 163 (100), 119 (12)	*p*-Coumaric acid isoprenyl ester (isomer)
**40**	33.86	339	269	MS^2[269]: 254 (100), 251 (75), 236 (40), 226 (15), 165 (35)	Unknown
**41**	34.68	280	369	MS^2[317]: 317 (100), 287 (37), 217 (6), 191 (25), 190 (56), 189 (34)	Pinobanksin-5-methyl-ether-3-*O*-pentanoate
**42**	36.37	268, 307	387	MS^2[387]: 387 (100), 293 (34), 281 (78), 267 (76), 255 (25)	*p*-Coumaric acid derivative
**43**	36.73	291	279	MS^2[279]: 235 (78), 195 (100), 162 (80), 118 (35)	*p*-Coumaric cinnamyl ester
**44**	37.05	294, 322	475	MS^2[475]: 433 (15), 415 (100), 400 (5), 365 (5), 253 (<1)	Pinobanksin-7-methyl-ether-5-*O*-*p*-hydroxyphenylpropionate
**45**	37.36	292	341	MS^2[341]: 253 (100)	Pinobanksin-3-*O*-butyrate or isobutyrate
**46**	37.78	292	353	MS^2[353]: 271 (1), 253 (100)	Pinobanksin-3-*O*-penteonate
**47**	38.42	259, 299	381 or 377	MS^2[377]: 359 (47), 335 (28), 333 (2), 319 (6), 317 (100), 315 (15), 273 (3), 264 (2)	Unknown
**48**	38.60	294, 322	399	MS^2[399]: 355 (39), 179 (100), 135 (50)	Caffeic acid derivative
**49**	39.51	283, 347	565	MS^2[565]: 455 (8), 417 (18), 283 (100), 269 (32)	*p*-Coumaric acid-4-hydroxyphenylethyl ester dimer

Table S1 (continued). Characterisation of phenolic compounds from Portuguese propolis by UHPLC-DAD-ESI-MSn.

Nr	t_R (min)	UV max	[M-H]$^-$ m/z	UHPLC/ESI-MS m/z (% base peak)	Compound
**50**	40.25	292	355	MS^2[355]: 271 (100)	Pinobanksin-3-*O*-pentenoate or 2-methylbutyrate
**51**	40.41	293	367	MS^2[367]: 271 (100), 253 (48); MS^3[271]: 253 (100), 225 (25), 151 (10)	Pinobanksin-*O*-hexenoate
**52**	40.58	322	319	MS^2[319]: 275 (100), 259 (34), 205 (15), 189 (<1)	Unknown
**53**	41.16	322	295	MS^2[295]: 251 (66), 211 (49), 178 (100), 134 (66)	Caffeic acid cinnamyl ester (isomer)
**54**	41.29	322	315	MS^2[315]: 179 (100), 135 (30)	Caffeic acid derivative
**55**	42.12	290	369	MS^2[369]: 271 (14), 253 (100)	Pinobanksin-3-*O*-hexanoate
**56**	42.64	283	293	MS^2[293]: 275 (25), 249 (12), 209 (7), 197 (42), 185 (100), 149(9), 125(9)	*p*-Methoxy-cinnamic acid cinnamyl ester
**57**	42.72	295, 322	323, [M+46]$^-$:369	MS^2[323]:	Caffeic acid derivative
**58**	43.18	276	293	MS^2[293]: 275 (25), 249 (12), 209 (7), 197 (42), 185 (100), 149(9), 125(9)	*p*-Methoxy-cinnamic acid cinnamyl ester (isomer)
**59**	43.37	275	429 or 383		Unknown
**60**	45.66	289	315	MS^2[315]: 297 (30), 271 (20), 259 (3), 228 (5), 205 (100)	Unknown
**61**	45.67	311	473, [M+46]$^-$:519	MS^2[473]:	p-Coumaric acid derivative
**62**	48.56	273, 283,307	365	MS^2[365]: 365 (100), 239 (15), 228 (96), 213 (21), 174 (24)	Unknown

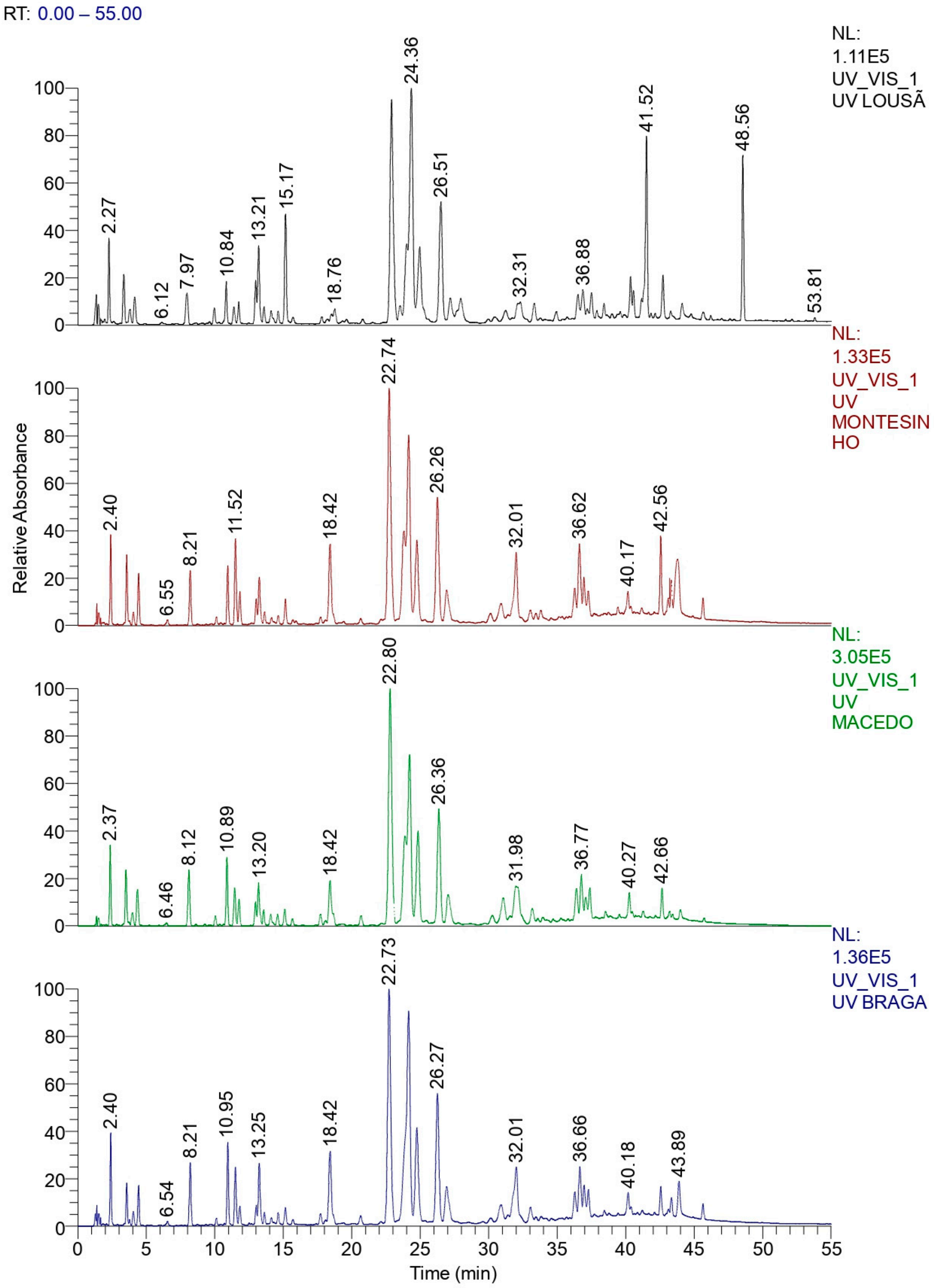

Figure S1. Chromatograms with UV detector (280 nm) of the four propolis samples.
